# A Computational Pipeline for the Extraction of Actionable Biological Information From NGS-Phage Display Experiments

**DOI:** 10.3389/fphys.2019.01160

**Published:** 2019-09-24

**Authors:** Antonios Vekris, Eleftherios Pilalis, Aristotelis Chatziioannou, Klaus G. Petry

**Affiliations:** ^1^UMR 1049 and U1029, INSERM, Bordeaux, France; ^2^Metabolic Engineering and Bioinformatics Program, Institute of Chemical Biology, National Hellenic Research Foundation, Athens, Greece; ^3^eNIOS Applications P.C., Athens, Greece

**Keywords:** phage display, Galaxy platform, enrichment analysis, network analysis, biological interpretation, Reactome, Gene Ontology

## Abstract

Phage Display is a powerful method for the identification of peptide binding to targets of variable complexities and tissues, from unique molecules to the internal surfaces of vessels of living organisms. Particularly for *in vivo* screenings, the resulting repertoires can be very complex and difficult to study with traditional approaches. Next Generation Sequencing (NGS) opened the possibility to acquire high resolution overviews of such repertoires and thus facilitates the identification of binders of interest. Additionally, the ever-increasing amount of available genome/proteome information became satisfactory regarding the identification of putative mimicked proteins, due to the large scale on which partial sequence homology is assessed. However, the subsequent production of massive data stresses the need for high-performance computational approaches in order to perform standardized and insightful molecular network analysis. Systems-level analysis is essential for efficient resolution of the underlying molecular complexity and the extraction of actionable interpretation, in terms of systemic biological processes and pathways that are systematically perturbed. In this work we introduce PepSimili, an integrated workflow tool, which performs mapping of massive peptide repertoires on whole proteomes and delivers a streamlined, systems-level biological interpretation. The tool employs modules for modeling and filtering of background noise due to random mappings and amplifies the biologically meaningful signal through coupling with BioInfoMiner, a systems interpretation tool that employs graph-theoretic methods for prioritization of systemic processes and corresponding driver genes. The current implementation exploits the Galaxy environment and is available online. A case study using public data is presented, with and without a control selection.

## Introduction

Phage Display has been widely used to select peptides binding to a variety of targets, *in vitro* or *in vivo*, with complexities ranging from a single macromolecule (Rodi and Makowski, 1999; Bábíčková et al., 2013) to diffuse pathological lesions (Pasqualini and Ruoslahti, 1996). Peptides identified using this technique have been successfully used for specific site drug delivery and *in vivo* imaging (Deutscher, 2010). The complexity of the selected repertoires of peptides is a function of the complexity of the target. Complex selections were poorly analyzed before the introduction of Next Generation Sequencing (NGS), which offered a detailed view of the peptide sequences (Dias-Neto et al., 2009). Software solutions were developed to compare the contents of several repertoires to identify common or specific sequences (Kolonin et al., 2006). In parallel, the hypothesis of mimicked proteins was advanced, based on the assumption that some peptides share sequence similarity with protein domains, and thus mimic the physiological interaction of the protein domain with its targets. In this scope, sequence comparison was usually performed using available tools, performing probabilistic (BLAST) (Altschul et al., 1990) or best-match (Needleman–Wunsch) (Smith and Waterman, 1981) mappings. A tool more adapted to analysis of phage display data, named PepTeam was developed by Hume et al. (2013), based on an algorithm producing all the ungapped matches of the peptides of a repertoire, compared against a set of proteins. Here we introduce PepSimili, a new computational tool significantly extending the capabilities of PepTeam and suitable for large-scale analysis of phage display data derived from NGS. PepSimili integrates the mapping function of PepTeam and extends the analysis with (a) an evaluation and subtraction of the local noise due to random mappings, (b) the subtraction of the signals produced by a control repertoire, and (c) filtering of derived proteins using a mapping score.

Moreover, PepSimili automatically manages a systems-level biological interpretation, in terms of underlying biological processes and master regulator genes. Pathway and functional analysis is performed by coupling the mapping functions of PepSimili with BioInfoMiner (Pilalis and Chatziioannou, 2013; Koutsandreas et al., 2016), an algorithm that performs systemic functional interpretation of the phenotype interrogated through the phage-display experiment. The interpretation algorithm performs by projecting the highest ranked proteins onto ontological and pathway networks with hierarchical structure. Highest ranked proteins are those presenting statistically significant accumulation of non-random peptide sequence matches. Their mapping on ontological and pathway networks enables the extraction of ranked lists of putative systemic processes and/or pathways, reflecting the underlying components involved in the manifestation of the investigated phenotype. The master regulatory genes and their respective protein products are ranked according to their contribution to the systemic processes.

Overall, PepSimili derives a systems-level interpretation of the mechanisms impacted by the cumulative effect of multiple mimicking peptides on protein networks. Ultimately, it manages to shortlist and rank candidate target proteins deriving from Phage Display experiments, according to their functional impact. The application is implemented on an instance of the Galaxy platform (Afgan et al., 2018). Through its user-friendly visual editor, the execution of the workflow is easily accessible to the basic user without prior experience in bioinformatics or in command-line oriented analyses. Additionally, the Galaxy platform already provides the tools necessary for the manipulation of the raw fastq files including quality filtering, trimming of the sequences to isolate the variable part of the recombinant phages and DNA to protein translation. PepSimili is the first application for the phage display technology implemented in Galaxy and which provides efficient mapping of short peptides on whole proteome databases. The tool is available online at http://pepsimili.e-nios.com:8080.

## Materials and Methods

### Workflow Implementation

**FIGURE 1:**
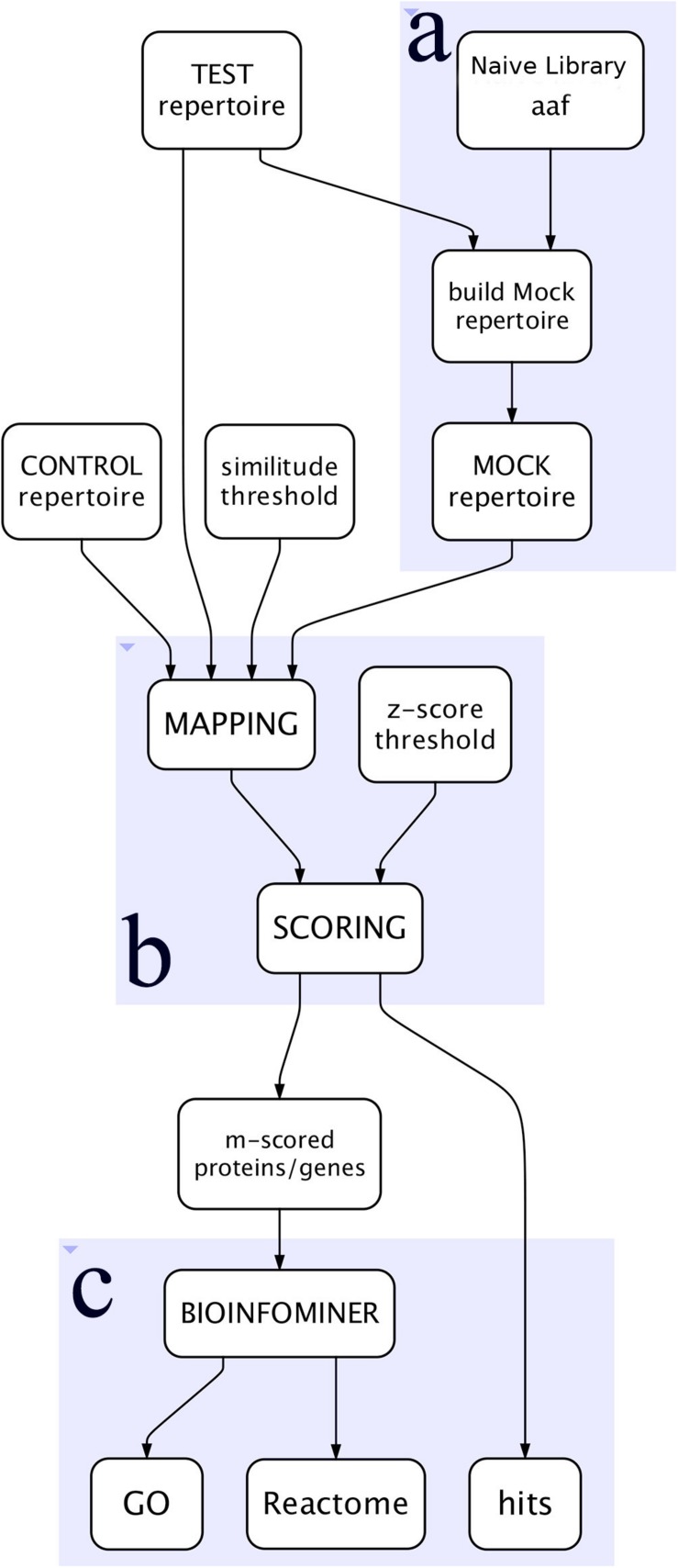
Simplified view of the workflow. **(A)** Represents the construction of the Mock repertoire, **(B)** the mapping and scoring processes, and **(C)** the BioInfoMiner analysis of the proteins/genes of interest.

The workflow, outlined in Figure 1, is written in Python language and implemented as a tool in a Galaxy cloud platform (Afgan et al., 2018).

### Inputs

PepSimili is presented as an integrated tool in Galaxy, accepting as inputs:

• A Test repertoire, containing the sequences of the peptides, with a length ranging from 5 to 15 residues, covering all commercially available phage display libraries (the most common being of 7–12 residues).
• A Control repertoire, if available. If not available, a file with a single poly-Tyr of the same length as the peptides (e.g., “WWWWWWW,” for *n* = 7) can be used.
• A table with the distribution of the amino acids (percentages) in the library used for the selections.
• A fasta file of the proteome, or a subset of proteins of interest.
• The threshold of similitude, h.
• The minimal z-score to be considered as significant for the selection of the outliers.
• The *p*-values and corrected *p*-values for BioInfoMiner.

### Workflow Steps

The main steps of the workflow are the following:

#### Calculation of the Amino Acid Frequencies of the Test Repertoire

The respective frequency of each amino acid in the library is calculated as a percentage.

#### Building of a Mock Repertoire

A Mock repertoire is built, composed of peptides of the same length and number (unique) as the peptides of the Test repertoire. Peptide sequences are quasi random, but respecting the amino acid frequencies of the phage library, as calculated in step 1. The Mock repertoire is used for the estimation of the noise produced by random mappings.

#### Mapping of Test and Control and Mock Repertoires on the Proteome

The problem of mapping a set of peptides on a set of proteins, respecting a threshold of similarity h, was previously addressed in Hume et al. (2013). Similarity between two peptides is evaluated using the PAM30 substitution matrix (Jones et al., 1992).

The algorithms, producing all the ungapped matches, of the peptides of a repertoire, on a set of proteins, were implemented in C++ and the code of the four modules necessary to produce the mappings and provide the resulting profiles is available at https://github.com/cbib/pepteam.

In total three mappings are performed, respectively for Test (T), Control (C) and Mock (M) repertoire (built in step 2). In the resulting files are reported the matching peptides, the similarity score and the matching position on the corresponding protein.

#### Signal Extraction

The mappings are used to produce a signal profile of mappings for each protein and for each of the T, C, and M repertoires. As signal profile is defined the sum of the hits in each amino acid position on the protein. The profile of background noise, as estimated from the Mock repertoire and representing random mappings, is subtracted from the signal profile of the Test repertoire, for each protein. If a Control repertoire is available, corresponding profiles are subtracted too, in order to extract a final signal profile representing meaningful peptide matches.

#### Scoring and Ranking of Proteins

After subtraction, the resulting signal profiles are used to generate a mapping score for each protein, termed m-score, which is the sum of the mappings from all positions, divided by the portion of the protein comprising at least one peptide match.

The distribution of the *m*-scores is calculated and each protein is annotated as *z*-score. The *z*-score cut-off set by the user (confidence level) is used to extract the list of proteins of interest for the next step of the analysis, which are thus outliers according to the calculated m-score distribution.

#### Systemic Biological Interpretation

Biological interpretation is performed for the set of promoted proteins from the previous step, using adapted algorithms from Chatziioannou and Moulos (2011), Moutselos et al. (2011), Pilalis and Chatziioannou (2013). The algorithm performs statistical and network analysis on controlled biological hierarchical vocabularies, here Gene Ontology (GO) (Ashburner et al., 2000) and Reactome pathways (Croft et al., 2014). This step (see section “Graph-Based Biological Interpretation,” below) derives significantly impacted biological processes and the respective driver genes linking these processes. It should be noted here that, with minimal programming effort, this algorithmic step can be adapted to exploit additional biological ontologies for network analysis.

### Outputs

The workflow produces as outputs:

• The signal profiles of the mapping of the peptides of the Test repertoire, showing the largest cluster of similar peptides, each peptide belongs to (#5).
• The signal profiles of the mappings of the Test, Mock and Control repertoires (#6, #7, and #8, respectively).
• The signal profiles of the mappings of the Test repertoire, minus both the Mock random mappings and the Control mappings (#9).
• Two files corresponding to the Test and Mock repertoires (#10 and #11, respectively) reporting all the mappings on each protein, indicating the position of the mapping, the homology of the peptide with the underlying protein segment, the peptide sequence, and three characteristics of the peptide: the number of occurrences in the repertoire, the number of peptides in the biggest cluster of similar peptides in the Test repertoire and the number of proteins on which the peptide is mapping.
• The list of proteins of interest, with z-scores above the chosen threshold (#12).
• The list of genes of interest, encoding the proteins of interest (#13), used as input for BioInfoMiner.
• The enriched GO terms characterizing the genes of interest (#14).
• The list of driver genes linking the processes pinpointed by the GO terms enrichment (#15).
• The list of the Reactome pathways, in which the genes of interest participate (#16).
• The list of the Reactome gene ranking (#17).

### Parameters

PepSimili is an integrated tool performing all steps of the analysis of the available repertoires in a single run. A group of satellite and intermediate scripts are also available in the platform, for access to intermediate steps of the workflow (listed in the tool menu as “PepSimili tools”).

#### Size of the Experimental Repertoires

The quality of the experimentally obtained peptide repertoires determines the mappings and thus the m-scoring. PepSimili uses the unique peptide sequences present in a repertoire to produce the mappings and calculate the m-scores. It is necessary to dispose of repertoires large enough to optimize the density of the mappings. A minimum of 40000 unique peptide sequences of 7mers is necessary. Usually NGS provides millions of reads for each repertoire, corresponding at hundreds of thousands of unique peptide sequences for targets as simple as cell cultures, thus covering the requirements of PepSimili.

For a visualization of the distribution of the *m*-score the script Sat_distri can be used; it produces a table with the distribution of the scores from any profile file.

Some experimental conditions may produce Test and Control repertoires of different sizes. If the Control repertoire does not fulfill the required equal size, it is advisable to complete by an equal number of unique sequences randomly chosen from the Test repertoire.

#### Amino Acids Distribution and Mock Repertoire

The distribution along the proteins of the mappings is sensitive to the frequencies of the amino acids of each repertoire (aaf), depending on local distributions. To generate a Mock repertoire allowing an adapted evaluation of the local random noise, it is necessary to apply an amino acid distribution as the one observed in the library being used for the selections. Most of the libraries are constructed using NNK codons, and the distribution of codons and amino acids is further distorted during the amplification of the library. Usually this information is available for commercially available libraries. For custom constructs it is necessary to include a sample of the library in the NGS experiments and calculate the aaf table. The script Sat_aaf uses as input a peptides occurrences table and produces the corresponding amino acids frequencies file. At minimum, if no experimental information is available, it is advised to use the theoretical amino acids frequencies.

The influence of the amino acids frequencies on the mapping’s distribution is shown in Supplementary Figures 1, 2. PepSimili accepts as input aaf tables with frequencies expressed as percentages.

#### Similarity and h Threshold

The degree of similarity of two peptides is calculated using the PAM30 substitution matrix. Only positive values can be handled by PepSimili, ranging from 0 to 1. For 7mers we recommend a threshold h between 0.4 and 0.8, depending on the desirable degree of similarities. As the evaluation of the random mappings is made using the same stringency and this background noise is systematically subtracted from the Test repertoire signals, high stringencies are not obligatory. On the contrary, when the Test repertoire contains a low number of unique sequences, it is advisable to decrease the threshold h, in order to accordingly increase the density of the mappings and obtain a more suitable m-score distribution, for the selection of the proteins of interest. The influence of the choice of the h threshold on the profiles is shown in Supplementary Figure 3.

#### Confidence Level Threshold (*Z*-Score)

The distribution of the m-scores is the function of the number of peptides in the repertoires and the threshold h chosen for the analysis. When both the random and the control (non-specific ones) profiles are subtracted from the test profiles, theoretically all remaining signal is significant and there is no need to focus on outliers of the distribution of the m-scores (Supplementary Figure 4). However, it is advisable to approach the Y = aah; described system by the selection(s) starting with proteins presenting the relatively highest m-scores, and the default *z*-score threshold of 2.58 usually selects sufficient numbers of proteins to build a first overview of the system under study, using BioInfoMiner’s outputs. A threshold as low as 1.5 is still significant given the distribution of the *m*-scores.

### Limitations

#### Proteomes and Fasta File Header Format

It is mandatory to use a particular header format for the proteins fasta file, so the script correctly extract the gene symbols for biological interpretation: >ENSGXXXXXXXX| ENSTXXXXXX| Gene Symbol| ENSPXXXXXXXX. Such files can be obtained from BioMart (Smedley et al., 2015), using a simple query with, as filters, Gene stable ID, Transcript stable ID, Gene symbol and Protein stable ID. Two sets of human proteins are already included, one of general use (proteome 20k) and one minimal (proteome 10k), more restricted to interaction molecules, adapted for selections performed *in vivo* or on cells accessible via the blood vessels (e.g., endothelial cells such as HUVEC). These proteins belong in either of the following three classes: plasma membrane, extracellular matrix, or secreted proteins, and were selected based on their annotation with GO terms Cellular localisation (GO:0051641) and Extracellular Region Part (GO:0044421), in addition to the proteins of the human plasma proteome, taken from the Human Plasma Peptide Atlas (Schwenk et al., 2017). For each protein isoform, the most complete in terms of exons was included.

However, the user can upload and use any proteome respecting the above-mentioned header format and perform manually the biological interpretation, using the BioInfoMiner module available on the PepSimili server. The functional analysis currently supports *Homo sapiens*, *Mus Musculus*, *Rattus norvegicus*, *Gallus gallus*, *Sus scrofa*, *Danio rerio*, *Drosophila melanogaster*, *Caenorhabditis elegans*, and *Arabidopsis thaliana*.

#### Test Repertoire Size

We mentioned the necessity of a Test repertoire with more than 40000 unique sequences in order to generate optimal results from our tool. It is possible to generate partial results for smaller repertoires, which are often obtained for relatively simple target systems (assuming fewer binding sites). A satellite script named Sat_scoring is provided, using as input a profile file, preferably the T-M-C one, and producing a table of *m*-scored proteins.

### Graph-Based Biological Interpretation

BioInfoMiner algorithm uses protein annotations and ontologies as a starting point for functional and pathway analysis with statistical and graph-theoretic methods (Chatziioannou and Moulos, 2011; Moutselos et al., 2011; Pilalis and Chatziioannou, 2013; Koutsandreas et al., 2016). The algorithm comprises two main steps:

#### Ontological Process and Pathway Prioritization

The algorithm employs a combination of a parametric (Hypergeometric) and a non-parametric statistical test (bootstrap resampling). Initially the Hypergeometric test is used to assess the over-representation and initial ranking of annotation terms in the input gene list. This ranking is corrected by performing bootstrapping as an alternative to multiple test correction methods (Bonferroni, FDR), thus avoiding false assumptions about the distribution of *p*-values. Instead of adjusting the *p*-values, the bootstrapping algorithm reorders the initial distribution and prioritizes the less frequently observed enrichments which tend to represent broader pathways or functions and, thus, are of stronger biological content (Pilalis and Chatziioannou, 2013; Pilalis et al., 2015).

#### Gene/Protein Prioritization

Gene prioritization is performed by a graph-theoretical approach that exploits an ontological direct acyclic graph structure to detect and rank genes according to their impact as linkers in the topology of that graph (Moutselos et al., 2011; Koutsandreas et al., 2016), using semantic measure techniques. As background graphs, are used variations of the following ontologies and hierarchical pathways, corrected for inconsistencies (annotation bias, information content imbalance, gaps): Gene Ontology, Reactome, MGI Mammalian Phenotype Ontology (annotation of Human genes) and Human Phenotype Ontology (HPO).

The extracted ranked list of systemic processes and/or pathways, reflects the underlying components involved in the manifestation of the investigated phenotype, and provides a descriptive snapshot that links and integrates the examined individual genes into broader functional, indispensable modules that shape the cellular phenotype. The master regulatory genes and their respective protein products are ranked according to their contribution to the systemic processes.

## Results

### Galaxy-Based Tool for Integrated Analysis of Phage Display Data

The Galaxy instance of PepSimili is available at http://pepsimili.e-nios.com:8080. The tool is easily accessible in the left-hand menu (“Tools”), under the section *pepSimili*. There are two additional tool sections, *PepSimili tools* and *PepSimili subworkflows*, which comprise the collection of satellite/intermediary scripts and partial worklows for mapping repertoires and scoring proteins, respectively.

**FIGURE 2:**
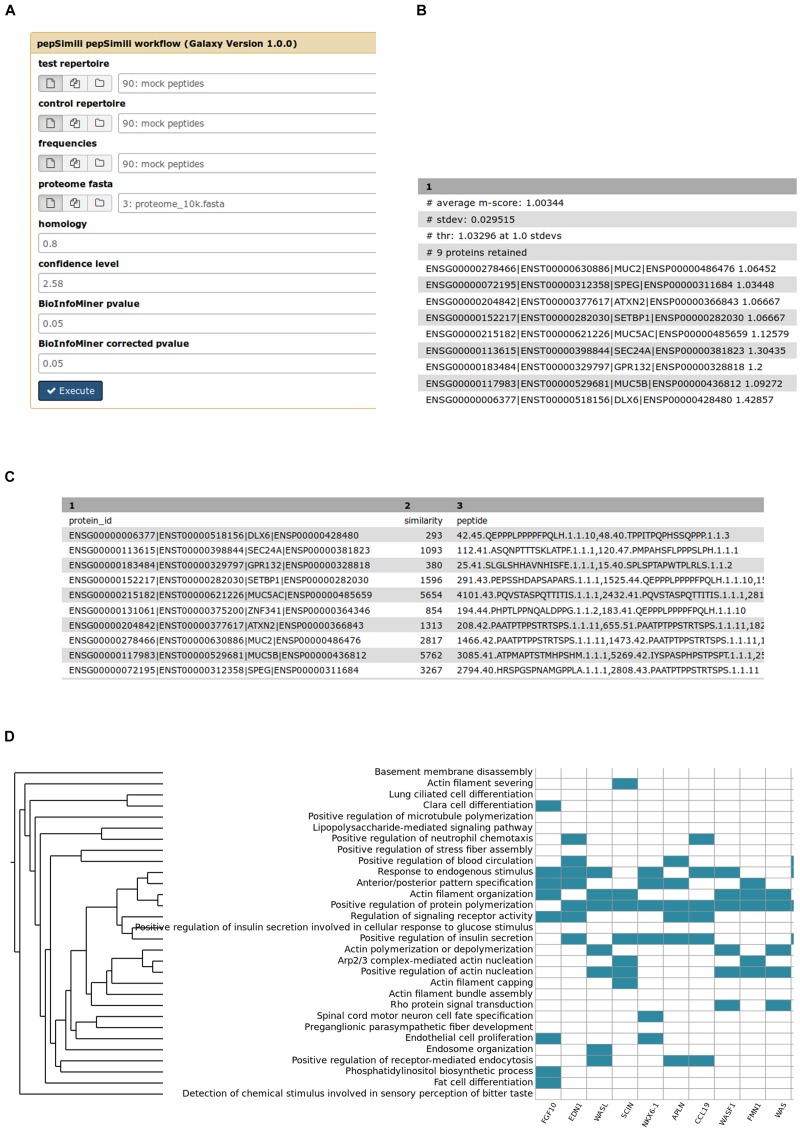
PepSimili main input and outputs. **(A)** The input form of the PepSimili automated workflow. Confidence level refers to the *z*-score cut-off, **(B)** output proteins, ranked by *m*-score, **(C)** output tabular file reporting the hits, **(D)** a heatmap visualizing the mapping of prioritized genes (*x* axis) to systemic processes (*y* axis).

PepSimili main input and outputs are shown in Figure 2. The tool accepts a Test and a Control repertoire, the amino-acid frequencies of the phage display library, the homology and confidence cut-offs and the *p*-values for the enrichment analysis (see section “Materials and Methods”) (Figure 2A). All steps are executed automatically, including the biological interpretation.

In Figures 2B,C are shown screenshots of, respectively, the output proteins with their m-scores, and an example of a file reporting peptide hits, including their headers, total hit similarity and the list of peptides matching the protein sequence, including additional details and metrics for each peptide (see Materials and Methods).

In Figure 2D is shown a heatmap, depicting the mapping of prioritized genes to systemic processes. The rank of each gene depends on the number of processes to which a gene participates. The more processes a gene is mapped to, the higher its rank is, highlighting its importance as a regulatory hub on the ontological network.

**FIGURE 3:**
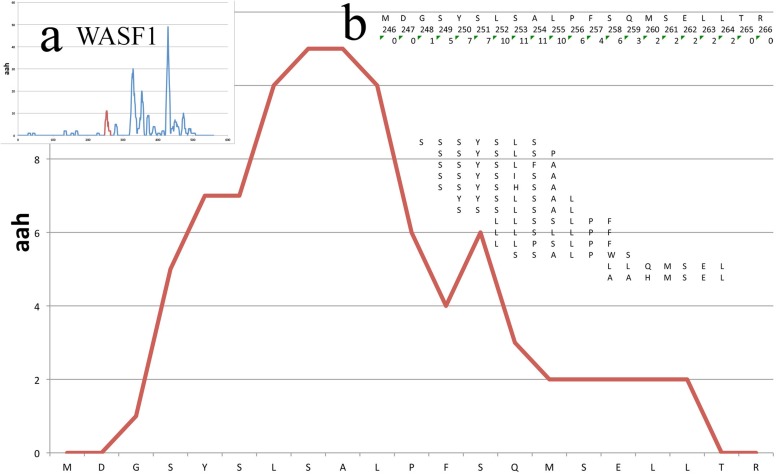
Mappings on a small portion of the WASF1 protein. The profile of the mappings of the HUVEC_tcm peptides (HUVEC under tumor conditioned medium) on the WASF1 protein is shown here, between residues 246 and 266. The *x*-axis presents the sequence, the *y*-axis the amino acid hits. Insert **(a)** shows the position of this particular stretch on the complete profile of the protein (red peek). Insert **(b)** shows in the first row the amino acid sequence, in second row the position of the amino acids and in third row the total aah per residue. Further below are shown the 13 peptides defining this stretch, positioned in frame with their similar sequence on the protein.

Figure 3 shows the peptide mappings on a segment of a protein (WASF1, see Case Study further below), also illustrating the calculation of the aah-score at each position, which is defined as the sum of the total peptide matches.

### Case Study

We present a case study using published data of phage display repertoires (Brinton et al., 2016). We used two of the described repertoires. Both were selected *in vitro*, on HUVEC cells that were cultured either in normal medium (Control) or in tumor conditioned medium (tcm) by tumoral cells (pancreatic adenocarcinoma, PDAC), which is serving as Test. The two samples of cultured cells were separately used for the biopanning with a combinatorial library of cyclic 7mers on phage display. In these studies, the recombinant portion of the pIII of the phage was amplified by PCR and used as template for deep sequencing. The aim of the study was to identify binders specific of the tcm-treated endothelial cells, expecting that their targets would be also expressed *in vivo* by cells in the microenvironment of cancerous tumors. Pepsimili extends the scope of the study to the identification of proteins with subsequences similar to the selected peptides. The full run is online available at http://pepsee.e-nios.com:8080/u/avek/h/example-huvectcm-vs-huvec.

Table 1 summarizes the genes prioritized by the BioInfoMiner algorithm using Gene Ontology and Reactome pathways. The gene prioritization results are shown in Supplementary Table 1 (GO) and Supplementary Table 2 (Reactome). Interestingly, the highest ranked genes include known markers of PDAC, such as WASL (Wiskott-Aldrich syndrome like) (Wei et al., 2012) and other WAS-associated proteins like WASF1, WASF2, WAS and WIPF1. Wiskott-Aldrich syndrome (WAS) is a rare X-linked primary immunodeficiency characterized by microthrombocytopenia, eczema, recurrent infections, and an increased incidence of autoimmunity and malignancies (Massaad et al., 2013). FGF10 induces migration and invasion in pancreatic cancer cells through interaction with FGFR2, resulting in a poor prognosis, thus FGF10/FGFR2 signaling is a promising target for new molecular therapy against pancreatic cancer (Nomura et al., 2008).

**TABLE 1:** Summary of the genes prioritized by BioInfoMiner (GO and Reactome).

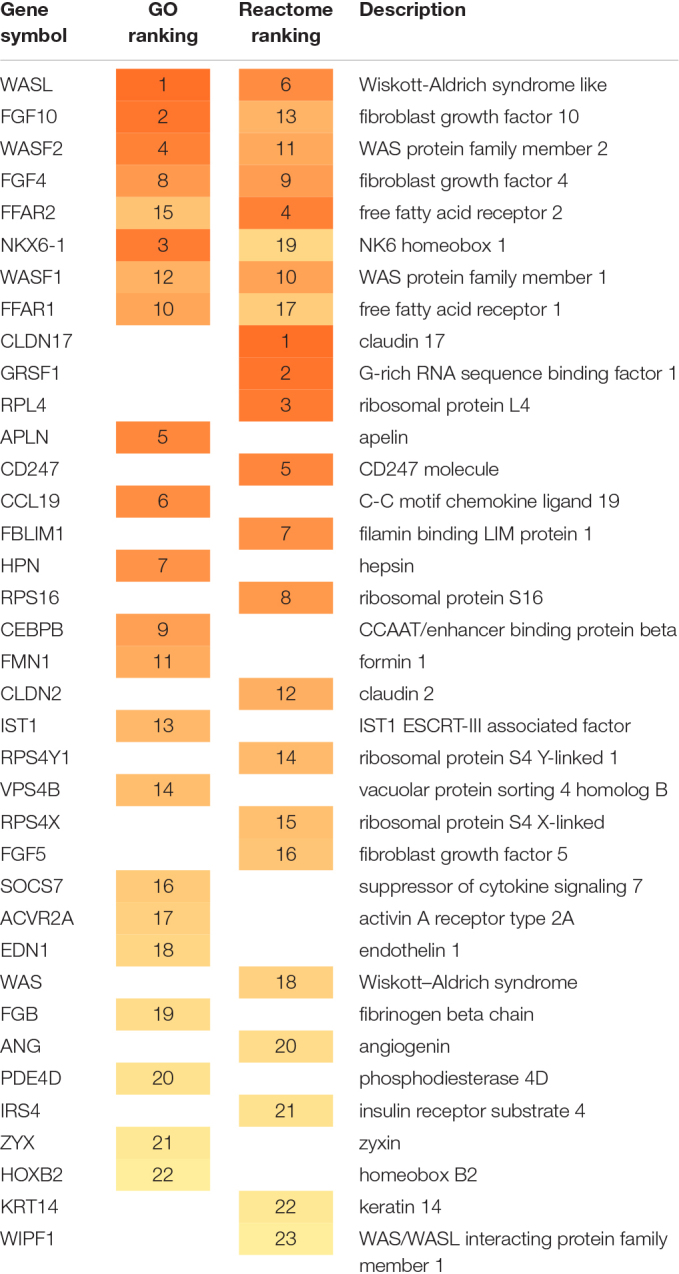

*In columns 2 and 3 the rank of each gene according to the GO terms and the Reactome pathways, respectively, is presented and color coded using a gradient from red to yellow. The table is sorted for increasing sum of the two ranks. Supplementary Tables 1, 2 correspond to the original outputs of BioInfoMiner.*

The main biological processes derived by BioInfoMiner (Supplementary Table 3) include Arp2/3 complex-mediated actin nucleation and actin polymerization, involved in multigeneration of dendritic protrusions for 3-dimensional cancer cell migration (Giri et al., 2013).

Prioritized Reactome pathways are shown in Supplementary Table 4. The results highlight activation of RHO GTPases, which results in formation of actin stress fibers, lamellipodia and filopodia through interaction with members of the Wiskott-Aldrich Syndrome Protein (WASP) (Vega and Ridley, 2008). In addition, the results indicate increased FGFR signaling, the inhibition of which achieved significant anti-cancer effects in pancreatic cancer (Zhang et al., 2014). Fukushima et al. (2015) showed that loss of free fatty acid receptor FFAR1 in pancreatic cancer cells promoted migration.

These results constitute an accurate and comprehensive interpretation of the underlying molecular complexity, describing the landscape of the molecular interactions captured by the set of mimicked proteins, derived from the Phage Display experiment. Supplementary Figures 5, 6 show the extracted networks, through projection on GO Biological Processes and Reactome Pathway hierarchical structures, respectively. The prioritized genes (Table 1 and Supplementary Tables 1, 2) constitute master regulators based on the topology of these networks, as they have a pivotal role in mediating the cross talking between distinct biological processes. This feature is illustrated in Supplementary Figure 7, which shows a more compact view of the projection of mimicked proteins on the GO corpus. Systemic processes were derived from semantic clustering of the enriched terms. The prioritized genes are regulators of distinct key processes underlying the PDAC pathology, such as Arp2/3 complex-mediated actin nucleation, Rho protein signal transduction, endothelial cell proliferation, endosome organization, fatty acid signaling and lipopolysaccharide signaling, neutrophil chemotaxis and microtubule polymerization. The oligopeptides mimicking the prioritized protein products can be easily retrieved through the Galaxy interface for further evaluation.

Overall, the present study showcases the capability of the integrative workflow for derivation and selection of biologically active peptides from complex Phage Display experiments, through effective filtering and comprehensive mapping of peptide repertoires on ontological networks and pathways.

## Discussion

Phage display coupled with NGS has been introduced almost 10 years ago, thus changing the way of how selected phage repertoires are perceived and analyzed. Deep sequencing techniques provide a global characterization of phage display libraries and selected repertoires, increasing the resolution depth and the potential of the phage display technology for the discovery of target molecules, through the identification of consensus motifs. Today, even the most complex repertoires of selected peptides, usually obtained by *in vivo* selections, can be sampled to obtain a detailed view of their composition and to monitor the progress of the enrichment of specific sequences after each selection/amplification cycle. NGS facilities are easily accessible by the experimentalist and can cover all the steps from the amplification of the DNA of the phages to the delivery of the raw reads in fastq format.

However, the development of analytical tools and strategies is far less advanced. Most software solutions have been in-house developed and not made widely available, by using standard, generic sequence comparison techniques, such as BLAST, or they had a limited scope such as PhastPep (Brinton et al., 2016) taking into account only identical sequences to compare repertoires.

Computational tools for Phage Display data analysis include RELIC (Mandava et al., 2004), PEPTIDE (Pizzi et al., 1995), SiteLight (Halperin et al., 2003), and SLiMFinder (Edwards et al., 2007), which enable sequence alignment and motif detection. However, these tools were designed for small-scale analyses, whereas deeper characterization emerged as a necessity with the advent of NGS techniques. For this purpose, newer methodologies have been developed for high throughput data processing and detection of consensus sequences (Fowler et al., 2011; Alam et al., 2015; Reich et al., 2015), although these techniques did not address the issue of selectivity and comparison among different physiological conditions. This particular problem was addressed by PHASTpep (Brinton et al., 2016), a MATLAB-based tool, which enables differential analysis and selection of peptides that discriminate among different cellular states. PepSimili combines selectivity and network-based functional analysis for prioritization of targets and derivation of biomarkers.

In addition, little has been done to help identifying the spectrum of proteins that are potentially mimicked by the plethora of selected peptides, and to aid in the elucidation of the biological circuits, on which the selection is made. Such information is particularly interesting for biopanning performed in tissues or *in vivo*, where the complexity of the obtained repertoires reflects the complexity of the biological process under investigation.

In this work, we present a novel strategy based on the identification of proteins containing regions similar to selected peptides obtained by phage display screening. Through their binding to their targets, these peptides are supposed to mimic functional domains of the identified proteins and protein regions. Indeed, most phage displayed peptide selections are originally intended to be used to analyze the repertoires resulting from screenings in complex systems, at least as complex as cell cultures. The presented strategy integrates furthermore the analysis of the retained proteins into a biologically meaningful signal. In contrast to other approaches, PepSimili does not take into account, in the first steps of analysis, the abundance of individual peptides in the repertoires. This is due to the fact that this metric can be greatly affected by the bias of preferential replication of a phage during amplification and the abundance of the target in the system under study, both factors that minimize the interest of its use. However, the abundance of each peptide is reported for mere convenience, in the final results.

Computational derivation of a set of proteins, with domains mimicked by the peptides, can also be helpful for the identification of the targets against which the peptides were selected, while studying their natural binders, as described in interactome databases. In complex systems, for which the peptides or protein domains were identified by our analysis, with the intention to be used as targeting tools for the homing of either therapeutic molecules or imaging agents, it is important to exclude those reacting with targets on healthy tissues. Usually, experimental strategies include the selection of peptides in a system as close as possible to the Test system, to produce a Control repertoire. In the example presented here, the Test system being endothelial cells cultured in tumor conditioned medium, is compared to the Control system being the same cells cultured in fresh medium. Such experimental design favors the identification of targeting molecules that would be specific of the Test system and absent from the Control. Obviously *in vivo* selections are preferable, in order to take into account all potential binding sites of the target molecules, being however far from trivial to be performed, in many cases.

Any set of random peptides presents similarities with the peptides of a set of proteins. It is, therefore, important to be able to evaluate this background noise, which sets an important informational bias confounding the interpretation, and subtract it from the signals obtained by a set of selected peptides, as shown in Supplementary Figure 4. In PepSimili, this background noise is systematically subtracted from the signal obtained by the Test repertoire. The approach is general enough and applicable to other high-throughput systems that generate massive peptide repertoires and thus necessitate systematic evaluation and elimination of the background noise. For instance, recently was reported an integrated bacterial system for the discovery of chemical rescuers of disease-associated protein misfolding, which enables massive screening of cyclic oligopeptides with potential pharmacological action against neurodegenerative diseases (Matis et al., 2017). In this system, large combinatorial libraries are biosynthesized in E. coli cells and simultaneously screened for their ability to rescue pathogenic protein misfolding and aggregation, using an ultrahigh-throughput fluorescence-based genetic assay. The high-throughput assay can generate combinatorial libraries of up to 10^8^ random peptide sequences. Eventually, coupled with deep sequencing for acquisition of the expressed sequences and *in vitro* validation (Matis et al., 2017), the system derives repertoires of potentially bioactive peptides orders of magnitude smaller (10^2^–10^4^). However, further *in silico* screening of the oligopeptide repertoires using PepSimili may, on one hand, dramatically reduce the number of candidate oligopeptides, and on the other hand provide a rational basis for peptide selection based on their functional interpretation in terms of impacted biological mechanisms.

Importantly, our methodology enables a systems-level interpretation, through streamlined mapping of the selected mimicked proteins to ontological and pathway networks, providing actionable insights. The BioInfoMiner module derives a small set of orthogonal, systemic processes, accompanied by the respective master regulatory genes linked with a significant part of them, altogether constituting a biomarker signature with actionable potential for clinical, therapeutic or diagnostic processes. Our study demonstrates the efficacy of this integrative workflow using public Phage Display data. Indeed, the tool automatically derived and prioritized key regulators and systemic processes underlying the PDAC pathology. Another potential application area of our approach is the field of Metagenomics, where computational platforms are being constantly developed for analysis, management and annotation of large-scale sequence data (Kunin et al., 2008; Lugli et al., 2016; Koutsandreas et al., 2019). Metagenomic analyses generate massive sequence data, including large amounts of partial or incomplete peptide sequences, stressing the necessity for more efficient annotation methodologies. Our approach that combines massive mapping of peptides to functional networks may enable a more efficient interpretation of the genomic information in the metagenomics content.

Finally, PepSimili is presented in a user-friendly environment, Galaxy, as an integrated tool that performs a complete analysis at a push of a button. A collection of satellite scripts and workflows is also provided, to propose tentative discovery paths that can be followed to complement the actual results, intending to encourage power users to develop, and share with the community new tools/scripts, adding to our work. This implementation facilitates the development of future extensions of the workflow and, importantly, the adaptation of the methodology to other high-throughput technologies, as mentioned above.

## Author Contributions

AV and KP contributed to PepSimili conception. EP and AC contributed to BioInfoMiner conception and implementation. EP contributed to PepSimili integration. AV, EP, AC, and KP wrote and revised the manuscript.

## Conflict of Interest

EP and AC were employed by company eNIOS Applications P.C. The remaining authors declare that the research was conducted in the absence of any commercial or financial relationships that could be construed as a potential conflict of interest. The reviewer MV and the handling Editor declared their shared affiliation at the time of the review.
