# Supplementary material for: A Computational Pipeline for the Extraction of Actionable Biological Information From NGS-Phage Display Experiments
Source: Front Physiol. 2019 Sep 24;10:1160. doi: 10.3389/fphys.2019.01160 (PMC6769401; doi:10.3389/fphys.2019.01160)
Supplement: Supplementary file 1 [file Data_Sheet_1.PDF]

Supplementary Material

Tables

Rank	gene_symbol	Description	Systemic processes
1	WASL	Wiskott-Aldrich syndrome like	['GO:0051127', 'GO:0009719', 'GO:0007032', 'GO:0006661', 'GO:0048260']
2	FGF10	fibroblast growth factor 10	['GO:0051127', 'GO:0009719', 'GO:0045444', 'GO:0006661', 'GO:0061140']
3	NKX6-1	NK6 homeobox 1	['GO:0051127', 'GO:0032024', 'GO:0009719', 'GO:0045444']
4	WASF2	WAS protein family member 2	['GO:0051127', 'GO:0007265', 'GO:0009719', 'GO:0006661']
5	APLN	apelin	['GO:0051127', 'GO:0009719', 'GO:1902622', 'GO:0048260']
6	CCL19	C-C motif chemokine ligand 19	['GO:0051127', 'GO:0009719', 'GO:1902622', 'GO:0048260']
7	HPN	hepsin	['GO:0009719', 'GO:0034769', 'GO:0006661']
8	FGF4	fibroblast growth factor 4	['GO:0009719', 'GO:0006661', 'GO:0035137']
9	CEBPB	CCAAT/enhancer binding protein beta	['GO:0009719', 'GO:1902622', 'GO:0045444']
10	FFAR1	free fatty acid receptor 1	['GO:0051127', 'GO:0032024', 'GO:0009719']
11	FMN1	formin 1	['GO:0051127', 'GO:0045010', 'GO:0035137']
12	WASF1	WAS protein family member 1	['GO:0051127', 'GO:0007265', 'GO:0009719']
13	IST1	IST1, ESCRT-III associated factor	['GO:0051127', 'GO:0009719', 'GO:0007032']
14	VPS4B	vacuolar protein sorting 4 homolog B	['GO:0051127', 'GO:0009719', 'GO:0007032']

15	FFAR2	free fatty acid receptor 2	['GO:0051127', 'GO:0009719', 'GO:0045444']
16	SOCS7	suppressor of cytokine signaling 7	['GO:0009719', 'GO:0045444', 'GO:0006661']
17	ACVR2A	activin A receptor type 2A	['GO:0009719', 'GO:0045444', 'GO:0006661']
18	EDN1	endothelin 1	['GO:0051127', 'GO:0009719', 'GO:1902622']
19	FGB	fibrinogen beta chain	['GO:0051127', 'GO:0009719', 'GO:1902622']
20	PDE4D	phosphodiesterase 4D	['GO:0051127', 'GO:0009719', 'GO:1902622']
21	ZYX	zyxin	['GO:0051127', 'GO:0009719', 'GO:0006661']
22	HOXB2	homeobox B2	['GO:0009719', 'GO:0048483']

Supp. Table 1. List of genes prioritized by BioInfoMiner using Gene Ontology

Rank	gene_symbol	Description	Terms
1	CLDN17	claudin 17	['1500931']
2	GRSF1	G-rich RNA sequence binding factor 1	['192823']
3	RPL4	ribosomal protein L4	['192823']
4	FFAR2	free fatty acid receptor 2	['5658623']
5	CD247	CD247 molecule	['1500931']
6	WASL	Wiskott-Aldrich syndrome like	['1500931']
7	FBLIM1	filamin binding LIM protein 1	['1500931']
8	RPS16	ribosomal protein S16	['192823']
9	FGF4	fibroblast growth factor 4	['5658623']
10	WASF1	WAS protein family member 1	['1500931']
11	WASF2	WAS protein family member 2	['1500931']
12	CLDN2	claudin 2	['1500931']
13	FGF10	fibroblast growth factor 10	['5658623']
14	RPS4Y1	ribosomal protein S4, Y-linked 1	['192823']
15	RPS4X	ribosomal protein S4, X-linked	['192823']
16	FGF5	fibroblast growth factor 5	['5658623']
17	FFAR1	free fatty acid receptor 1	['5658623']
18	WAS	Wiskott-Aldrich syndrome	['1500931']
19	NKX6-1	NK6 homeobox 1	['5658623']

20	ANG	angiogenin	['1500931']
21	IRS4	insulin receptor substrate 4	['5658623']
22	KRT14	keratin 14	['1500931']
23	WIPF1	WAS/WASL interacting protein family member 1	['1500931']

Supp. Table 2. List of genes prioritized by BioInfoMiner using Reactome pathways

Biological Process

Rank	Term	Description	Enrichment	p-value	corrected p-value
1	GO:0051127	positive regulation of actin nucleation	6/13	6.511E-9	0.0011
2	GO:0008154	actin polymerization or depolymerization	6/44	1.914E-5	0.002
3	GO:0051125	regulation of actin nucleation	6/26	7.585E-7	0.0022
4	GO:0010469	regulation of signaling receptor activity	22/544	2.812E-6	0.0024
5	GO:0007015	actin filament organization	11/203	6.602E-5	0.0033
6	GO:2000601	positive regulation of Arp2/3 complex-mediated actin nucleation	3/7	6.820E-5	0.004
7	GO:0030838	positive regulation of actin filament polymerization	7/81	7.948E-5	0.0055

8	GO:0007032	endosome organization	6/71	2.903E-4	0.0059
9	GO:0048483	autonomic nervous system development	5/42	1.865E-4	0.006
10	GO:0030833	regulation of actin filament polymerization	8/146	5.894E-4	0.0077
11	GO:0032273	positive regulation of protein polymerization	7/111	5.592E-4	0.0079
12	GO:0032970	regulation of actin filament-based process	13/349	6.126E-4	0.0082
13	GO:0034769	basement membrane disassembly	2/3	4.760E-4	0.0088
14	GO:0032956	regulation of actin cytoskeleton organization	12/305	6.078E-4	0.0098
15	GO:0090022	regulation of neutrophil chemotaxis	4/35	9.800E-4	0.011
16	GO:0036257	multivesicular body organization	4/31	6.131E-4	0.0111
17	GO:1902624	positive regulation of neutrophil migration	4/33	7.814E-4	0.0113
18	GO:0071624	positive regulation of granulocyte chemotaxis	4/32	6.936E-4	0.0121
19	GO:0034315	regulation of Arp2/3 complex-mediated actin nucleation	3/16	1.002E-3	0.0123
20	GO:0051495	positive regulation of cytoskeleton organization	9/200	1.124E-3	0.0126
21	GO:0035137	hindlimb morphogenesis	4/37	1.212E-3	0.0151
22	GO:0030036	actin cytoskeleton organization	14/425	1.258E-3	0.0159

23	GO:0008064	regulation of actin polymerization or depolymerization	8/165	1.301E-3	0.0161
24	GO:0030832	regulation of actin filament length	8/166	1.352E-3	0.0164
25	GO:0032496	response to lipopolysaccharide	11/298	1.670E-3	0.0168
26	GO:1902905	positive regulation of supramolecular fiber organization	8/178	2.095E-3	0.019
27	GO:0060510	type II pneumocyte differentiation	2/5	1.560E-3	0.0195
28	GO:1902622	regulation of neutrophil migration	4/39	1.479E-3	0.0197
29	GO:0039702	viral budding via host ESCRT complex	3/20	1.964E-3	0.0204
30	GO:0009607	response to biotic stimulus	22/882	2.482E-3	0.0205
31	GO:0009617	response to bacterium	16/571	3.034E-3	0.0215
32	GO:0002237	response to molecule of bacterial origin	11/316	2.626E-3	0.0218
33	GO:0071622	regulation of granulocyte chemotaxis	4/47	2.961E-3	0.0225
34	GO:0032535	regulation of cellular component size	11/329	3.559E-3	0.0225
35	GO:0051493	regulation of cytoskeleton organization	14/476	3.504E-3	0.023
36	GO:0051704	multi-organism process	46/2348	3.105E-3	0.0231
37	GO:0097435	supramolecular fiber organization	12/379	3.704E-3	0.0247
38	GO:0032271	regulation of protein polymerization	8/195	3.659E-3	0.0252

39	GO:0048260	positive regulation of receptor-mediated endocytosis	4/51	3.986E-3	0.026
40	GO:0110053	regulation of actin filament organization	9/241	3.939E-3	0.0277
41	GO:0050673	epithelial cell proliferation	5/84	4.374E-3	0.0289
42	GO:0051014	actin filament severing	2/8	4.259E-3	0.0296
43	GO:0030041	actin filament polymerization	3/26	4.233E-3	0.0301
44	GO:0061140	lung secretory cell differentiation	2/9	5.430E-3	0.0318
45	GO:0046887	positive regulation of hormone secretion	6/129	6.221E-3	0.0324
46	GO:0030029	actin filament-based process	14/500	5.350E-3	0.0326
47	GO:0043207	response to external biotic stimulus	20/844	6.518E-3	0.0338
48	GO:0033993	response to lipid	20/837	5.975E-3	0.0339
49	GO:0071216	cellular response to biotic stimulus	7/175	7.276E-3	0.0339
50	GO:0090066	regulation of anatomical structure size	13/466	7.308E-3	0.035
51	GO:0021953	central nervous system neuron differentiation	7/173	6.850E-3	0.0359
52	GO:0051707	response to other organism	20/842	6.359E-3	0.0368
53	GO:0090277	positive regulation of peptide hormone secretion	5/95	7.330E-3	0.0373
54	GO:0021522	spinal cord motor neuron differentiation	3/33	8.319E-3	0.0378

55	GO:0046854	phosphatidylinositol phosphorylation	5/100	9.048E-3	0.0378
56	GO:0003002	regionalization	10/314	7.361E-3	0.0391
57	GO:0016050	vesicle organization	10/319	8.173E-3	0.0396
58	GO:1902903	regulation of supramolecular fiber organization	10/320	8.343E-3	0.0396
59	GO:0045444	fat cell differentiation	5/104	1.061E-2	0.0416
60	GO:0044089	positive regulation of cellular component biogenesis	13/491	1.095E-2	0.0418
61	GO:0007167	enzyme linked receptor protein signaling pathway	17/720	1.180E-2	0.0426
62	GO:0009952	anterior/posterior pattern specification	7/198	1.370E-2	0.0437
63	GO:0051047	positive regulation of secretion	11/391	1.219E-2	0.0444
64	GO:0071219	cellular response to molecule of bacterial origin	6/154	1.412E-2	0.0452
65	GO:0045823	positive regulation of heart contraction	3/39	1.318E-2	0.0459
66	GO:0009719	response to endogenous stimulus	28/1386	1.224E-2	0.0468
67	GO:0050913	sensory perception of bitter taste	3/41	1.509E-2	0.0471
68	GO:0007265	Ras protein signal transduction	6/160	1.674E-2	0.0485
69	GO:0045944	positive regulation of transcription by RNA polymerase II	23/1114	1.718E-2	0.0486
70	GO:1903524	positive regulation of blood	4/74	1.462E-2	0.0487

		circulation			
71	GO:0007169	transmembrane receptor protein tyrosine kinase signaling pathway	13/519	1.656E-2	0.0491
72	GO:0090596	sensory organ morphogenesis	8/255	1.687E-2	0.0491

Supp. Table 3. Biological Processes prioritized by BioInfoMiner using Gene Ontology

Rank	Term	Description	Enrichment	p-value	corrected p-value
1	5663213	RHO GTPases Activate WASPs and WAVEs	5/36	5.073E-5	0.003
2	5658623	FGFRL1 modulation of FGFR1 signaling	3/13	3.746E-4	0.0066
3	2033519	Activated point mutants of FGFR2	3/17	8.613E-4	0.0091
4	5654221	Phospholipase C-mediated cascade; FGFR2	3/18	1.025E-3	0.0119
5	444209	Free fatty acid receptors	2/5	1.239E-3	0.0158
6	190241	FGFR2 ligand binding and activation	3/20	1.408E-3	0.0214
7	2029482	Regulation of actin dynamics for phagocytic cup formation	6/118	2.283E-3	0.0233
8	5654700	FRS-mediated FGFR2 signaling	3/25	2.724E-3	0.0267
9	2428928	IRS-related events triggered by IGF1R	4/52	2.823E-3	0.0284
10	2428924	IGF1R signaling cascade	4/53	3.026E-3	0.0316

11	2404192	Signaling by Type 1 Insulin-like Growth Factor 1 Receptor (IGF1R)	4/54	3.240E-3	0.0363
12	210747	Regulation of gene expression in early pancreatic precursor cells	2/8	3.393E-3	0.0379
13	192823	Viral mRNA Translation	5/90	3.590E-3	0.045
14	1500931	Cell-Cell communication	6/130	3.691E-3	0.0492

Supp. Table 4. Pathways prioritized by BioInfoMiner using Reactome pathways

Figures

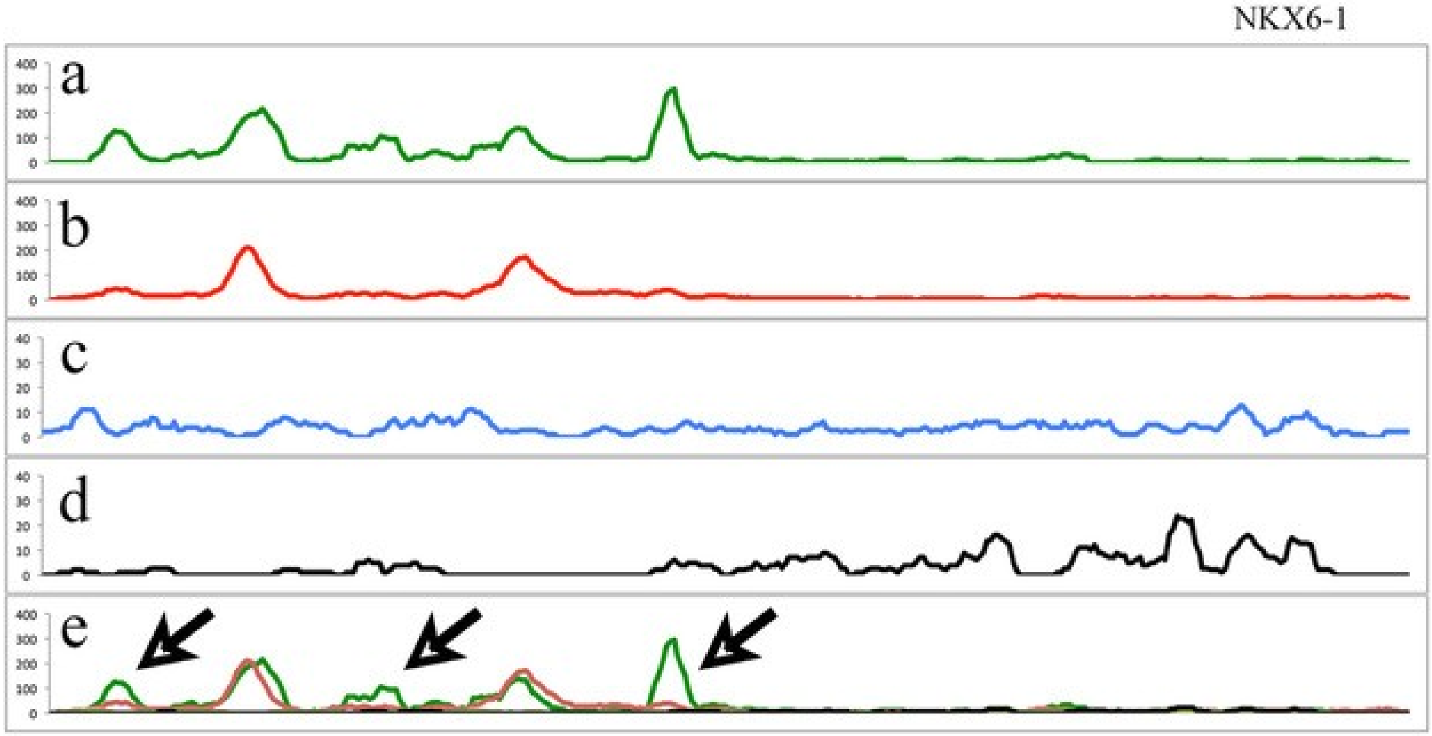

Supp. Fig. 1. Influence of the amino acids frequencies used to construct the Mock repertoire for the evaluation of the local noise due to random mappings. Mappings were made with a minimal h of 0.5. (a) profile by selected peptides (HUVEC_TMC); (b, c, d) profiles by mock repertoires with amino acid frequencies corresponding to the naive library, equal frequencies (5%) and scrambled frequencies of the naive library, respectively; (e) superposition of the above graphics. Y-axis for profiles (a, b, e) with a maximum of 400 hits, for profiles (c, d) with a maximum of 40. Arrows in profile (e) indicate domains with signals above the background noise

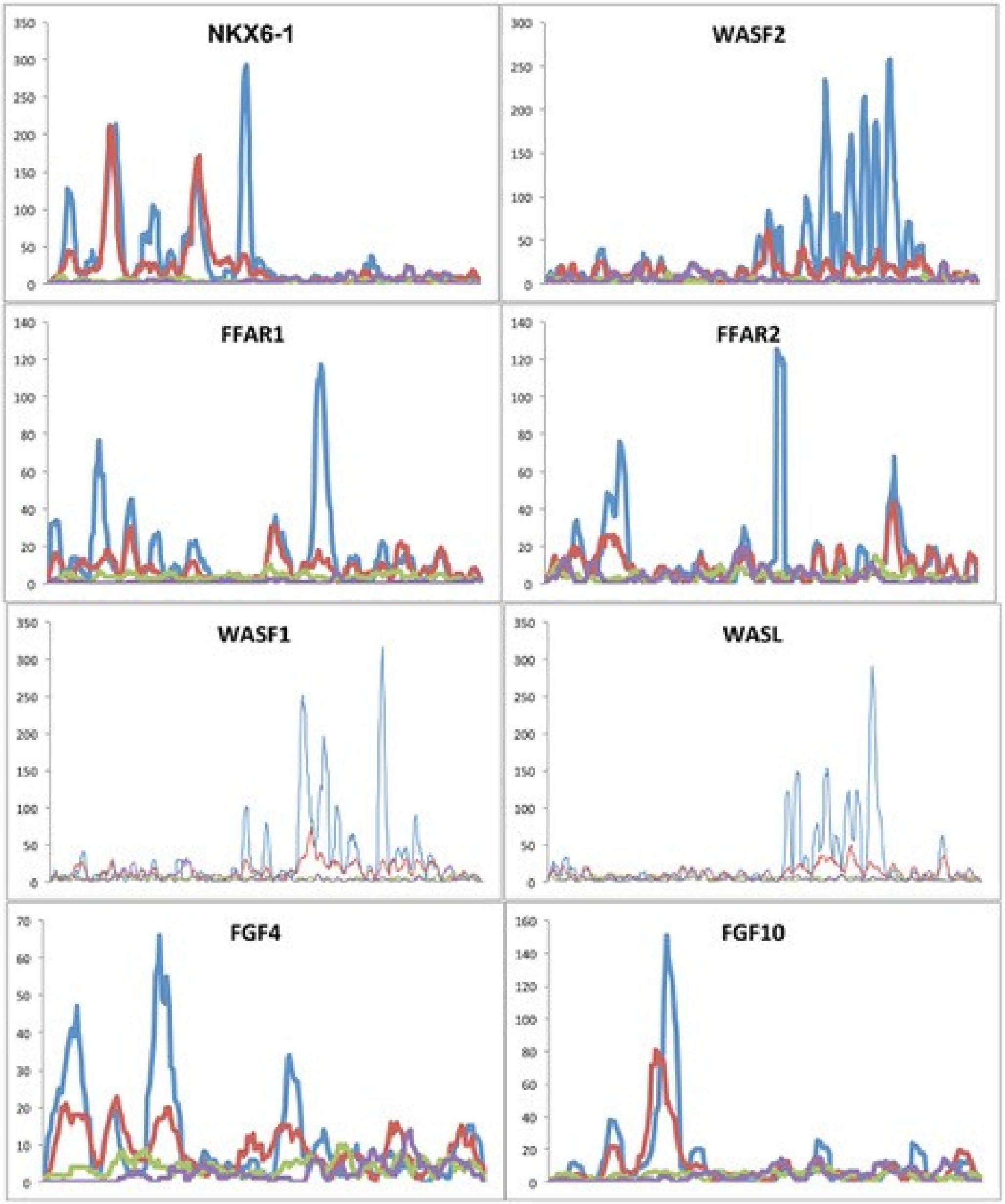

Supp. Fig. 2. Profiles of mappings on eight proteins. The same colour code as in supp. fig. 3 is used, with the four curves corresponding to (a-d) superposed as in (c). Mock repertoires with amino acid frequencies other than the ones of the naive library fail to provide an adequate evaluation of the background noise.

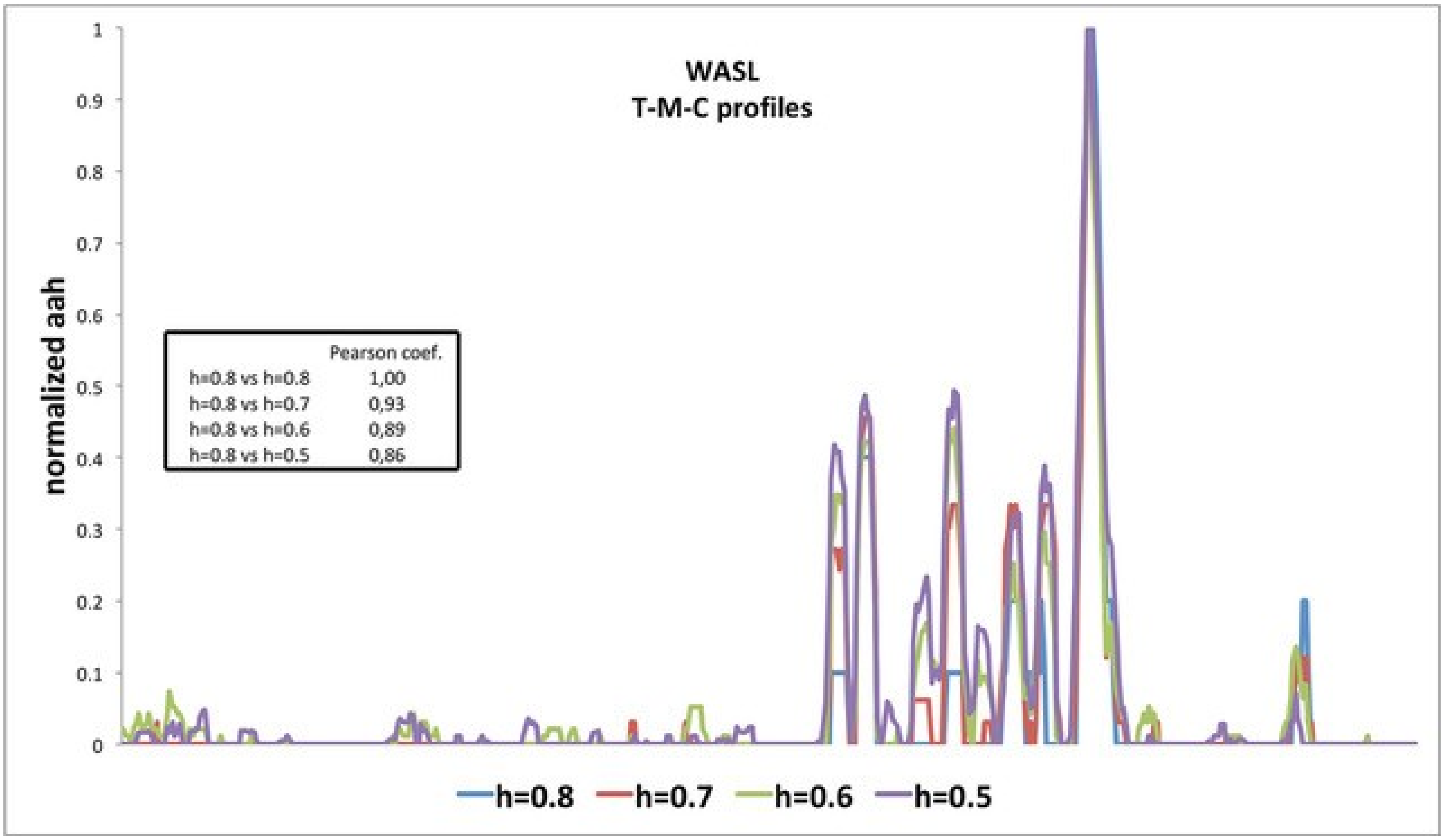

Supp. Fig 3. **Profile dependence on the similarity threshold (h).** The profiles of mappings of the HUVEC_TCM repertoire on the WASL encoded protein are presented here; four different values of h, from 0.5 to 0.8 were used. The profiles are normalised to the maximum aah value of each series for easier comparison. In insert the Pearson correlation coefficients between the most stringent profile (h=0.8) and the other three ones.

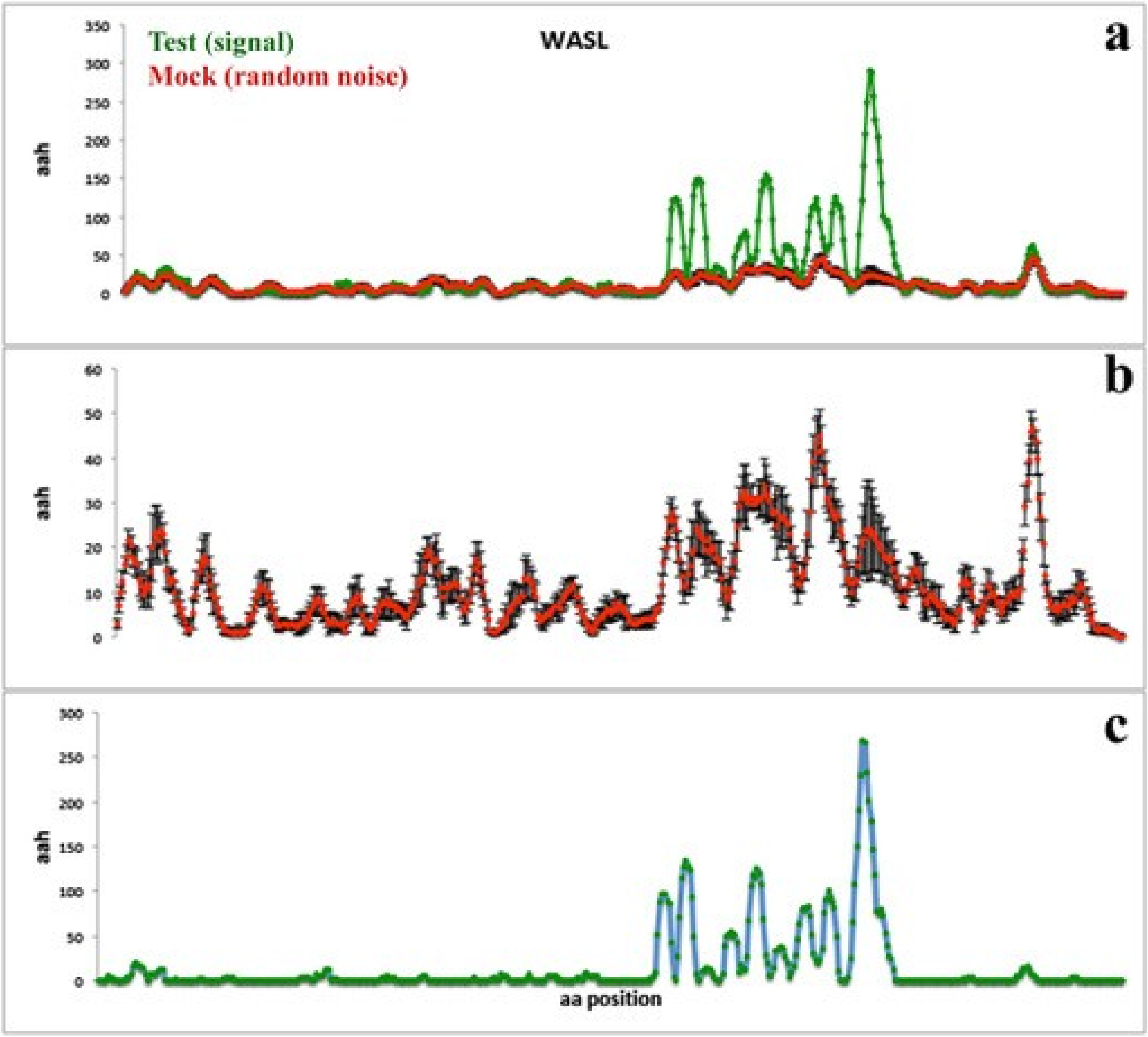

Supp. Fig. 4. Random mappings noise evaluation. The efficiency of the evaluation of the random noise by using a single mock repertoire is shown here. Mappings are made with a similarity threshold of h=0.5, for the HUVEC_TMC test repertoire and five independent mock repertoires. Panel (a) presents the superposition of the test profile and of the average of the five mock profiles. Panel (b) presents the average of the mock profiles with error bars corresponding to 1 standard deviation of the five independent profiling. Panel (c) corresponds to the signal after subtraction of the random mappings.

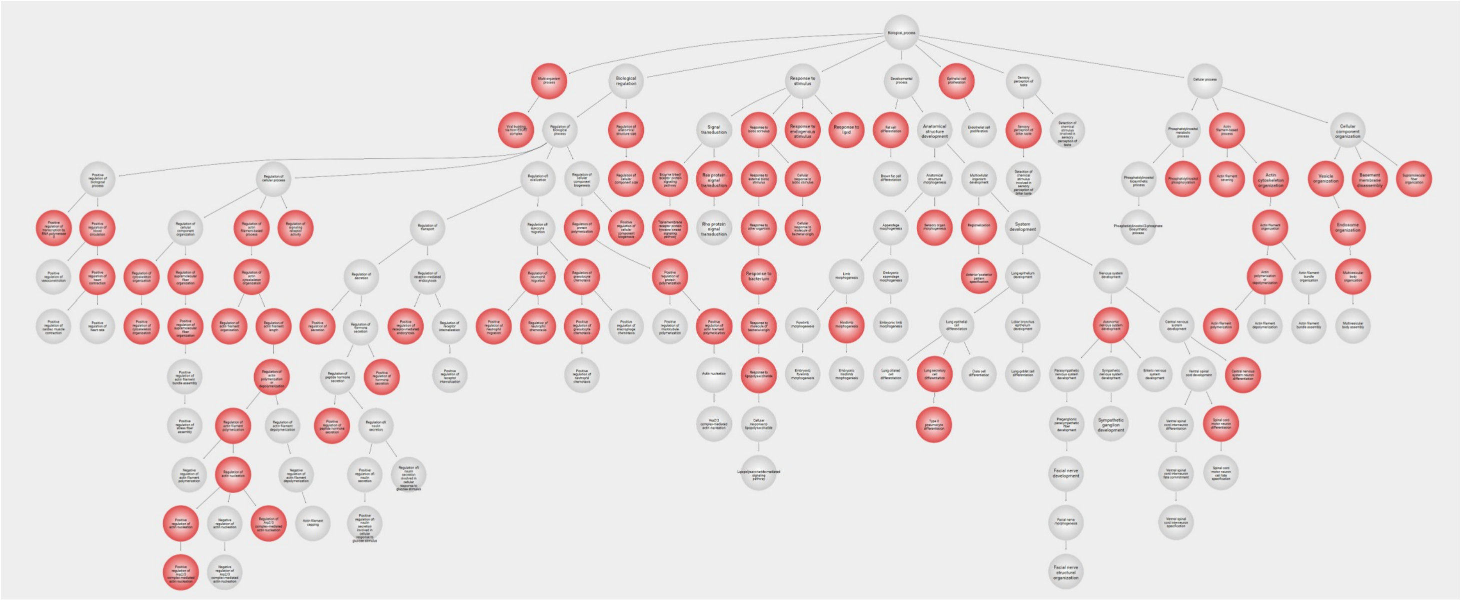

Supp. Fig. 5. Derived ontological network from BioInfoMiner analysis applied on Gene Ontology. The prioritized genes constitute master regulators based on the topology of these networks, as they have a pivotal role in mediating the cross talking between distinct biological processes.

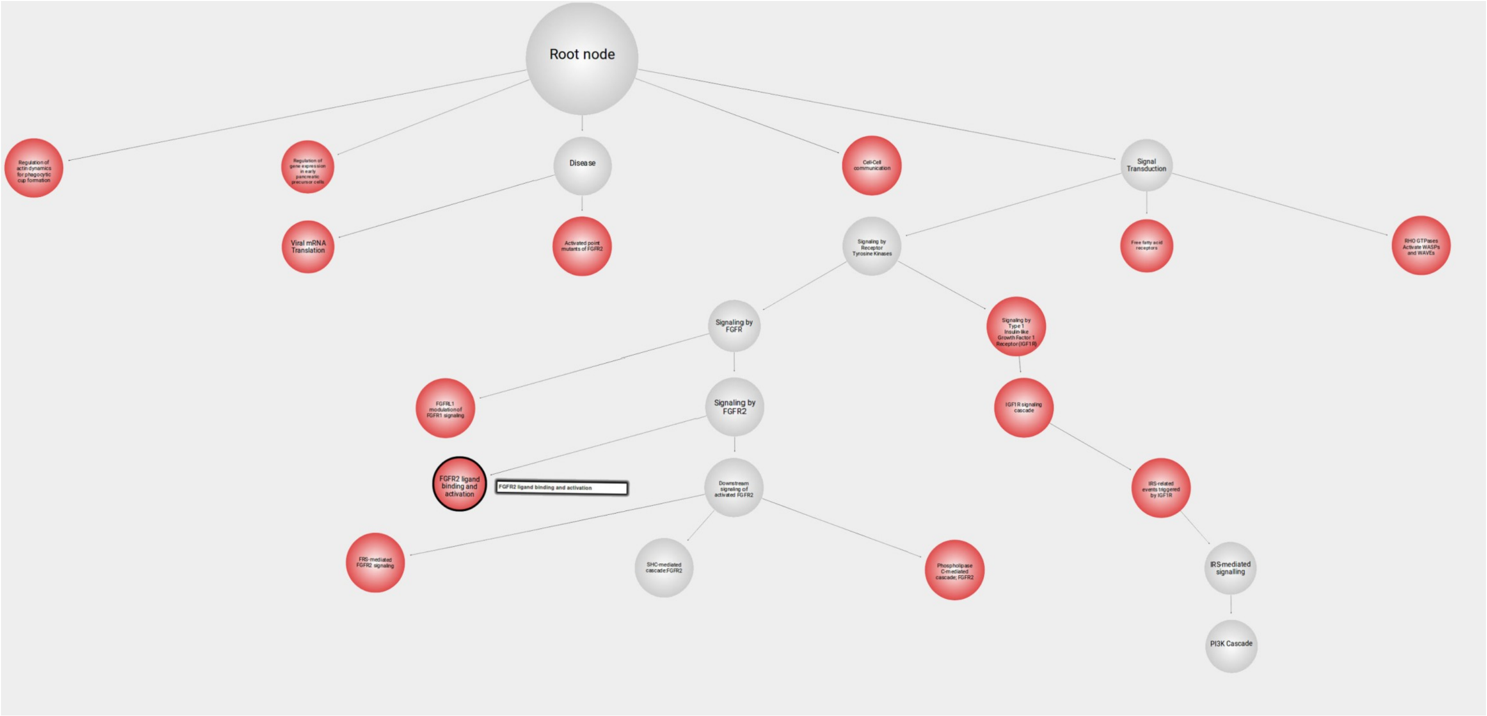

Supp. Fig. 6. Derived pathway network from BioInfoMiner analysis applied on Reactome Pathways. The prioritized genes constitute master regulators based on the topology of these networks, as they have a pivotal role in mediating the cross talking between distinct molecular pathways

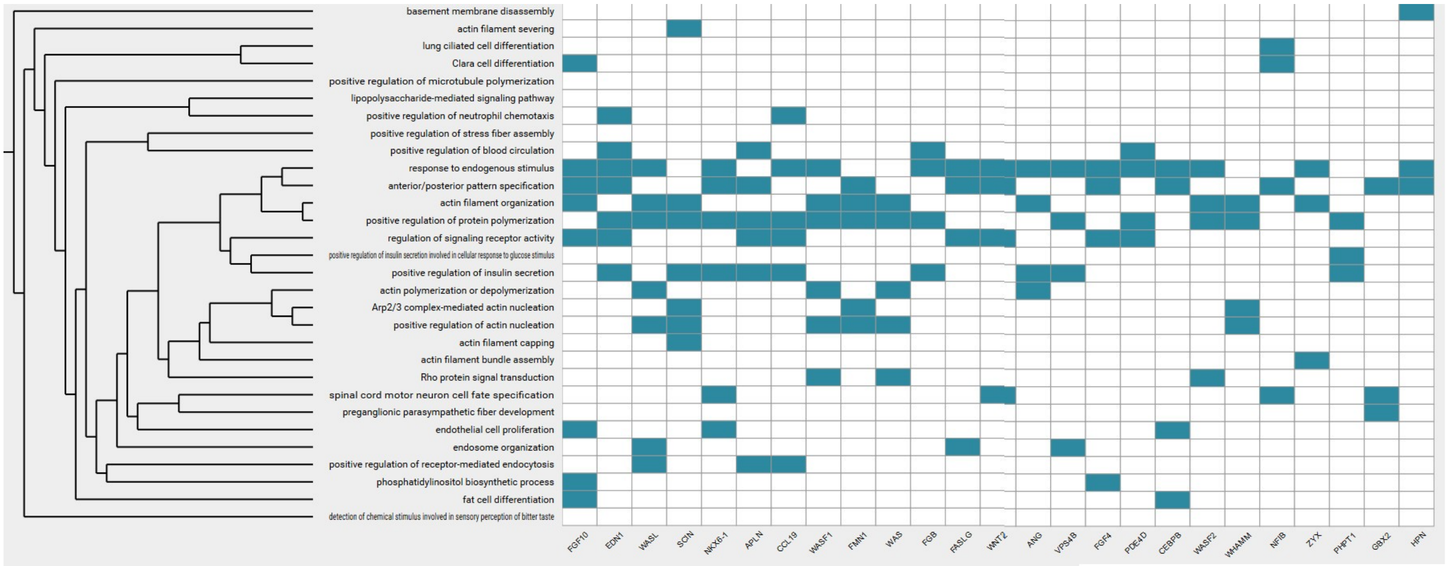

Supp. Fig. 7. Signature of systemic processes and master regulator genes derived from BioInfoMiner analysis on the phage display data. Systemic processes were derived from semantic clustering of the enriched terms. The regulatory effect of a gene to a systemic process is depicted by blue color. The prioritized genes are regulators of distinct key processes underlying the PDAC pathology
